# Reported handwashing practices of Vietnamese people during the COVID-19 pandemic and associated factors: a 2020 online survey

**DOI:** 10.3934/publichealth.2020051

**Published:** 2020-08-27

**Authors:** Le Thi Thanh Huong, Le Tu Hoang, Tran Thi Tuyet-Hanh, Nguyen Quynh Anh, Nguyen Thi Huong, Do Manh Cuong, Bui Thi Tu Quyen

**Affiliations:** 1 Environmental Health Department, Hanoi University of Public Health, Hanoi, Vietnam; 2 Biostatistics Department, Hanoi University of Public Health, Hanoi, Vietnam; 3 Vietnam Health Environment Management Agency, Ministry of Health, Hanoi, Vietnam

**Keywords:** COVID-19 pandemic, handwashing practices, associated factors, Vietnam, online survey

## Abstract

COVID-19 pandemic currently affects nearly all countries and regions in the world. Washing hands, together with other preventive measures, to be considered one of the most important measures to prevent the disease. This study aimed to characterize reported handwashing practices of Vietnamese people during the COVID-19 pandemic and associated factors. Kobo Toolbox platform was used to design the online survey. There were 837 people participating in this survey. All independent variables were described by calculating frequencies and percentages. Univariate linear regression was used with a significant level of 0.05. Multiple linear regression was conducted to provide a theoretical model with collected predictors. Seventy-nine percent of the respondents used soap as the primary choice when washing their hands. Sixty percent of the participants washed their hands at all essential times, however, only 26.3% practiced washing their hands correctly, and only 28.4% washed their hands for at least 20 seconds. Although 92.1% washed hands after contacting with surfaces at public places (e.g., lifts, knob doors), only 66.3% practiced handwashing after removing masks. Females had better reported handwashing practices than male participants (OR = 1.88; 95% CI: 1.15–3.09). Better knowledge of handwashing contributed to improving reported handwashing practice (OR = 1.30; 95% CI: 1.20–1.41). Poorer handwashing practices were likely due, at least in part, to the COVID-19 pandemic information on the internet, social media, newspapers, and television. Although the number of people reported practicing their handwashing was rather high, only a quarter of them had corrected reported handwashing practices. Communication strategy on handwashing should emphasize on the minimum time required for handwashing as well as the six handwashing steps.

## 1. Introduction

Coronavirus disease (COVID-19) is an infectious disease caused by a newly discovered coronavirus. Since its emergence in Wuhan, China in December 2019, severe acute respiratory syndrome coronavirus 2 (SARS-CoV-2) had rapidly spread throughout the world following an exponential growth curve prompting it to be characterized as a pandemic by the World Health Organization on March 11, 2020 [1],[2]. As of 12.00 pm on April 12, 2020, 1,780,315 cases of Coronavirus disease 2019, including 108,828 deaths, had been reported worldwide by more than 200 countries and territories [3]. Clinical presentations of COVID-19 may take up to 14 days to appear after exposure to the virus with the symptoms range from mild illness to severe pneumonia, and other life-threating complications which may lead to death [4],[5]. In Vietnam, a lower-middle-income country with the population of more than 96 million people, the first COVID-19 case was reported on 23^rd^ January 2020. As of 12^th^ April 2020 there were 258 infections, 144 of the affected patients have recovered and 0 death [3]. However, the threat of spreading COVID-19 in Vietnam is predicted to increase due to unidentified sources of infection in the community.

Hand hygiene is considered as one of the most fundamental preventive measures against many infectious diseases. Several studies have indicated that the efficacy of keeping hands clean reduced significantly the rates of infectious illnesses in the community, including diarrhea [6]–[9], cholera [7],[10]–[12], and pneumonia [6]. Besides, poor handwashing may contribute greatly to the risk of foodborne illness [13]. However, handwashing has remained an infrequent practice, especially for those who live in the least developed countries [14]. Eighteen percent of the global population (approximately 1.4 billion) had no basic handwashing facilities with soap and water available at home in 2017 [14]. A recent study conducted in 2019 in Northern mountainous provinces and the Central Highlands of Vietnam indicated that only 10% of the studied population performed hand hygiene with soap and water after defecation and this number increased to 54% after intervention activities were implemented [15].

World Health Organization (WHO) and the Vietnam Ministry of Health (MOH) has repeatedly emphasized that regularly and thoroughly performing hand hygiene with soap and water or alcohol-based hand rub is an effective way to prevent the spread of the new coronavirus SARS-CoV-2 [16],[17]. This virus spreads primarily through respiratory droplets when an infected person coughs or sneezes, or close personal contact, such as touching a contaminated surface and then touching eyes, noses or mouths before handwashing [5]. Therefore, promoting proper hand hygiene practices is a well-known preventive measure to control COVID-19 since effective treatment drugs and vaccines for COVID-19 are not currently available.

Widely reporting of statistics and facts about the COVID-19 pandemic by the global media may lead to confusion and anxiety about the disease along with the preventive measures, including handwashing recommended by many organizations including WHO and MOH. This study describes the knowledge and practices of people in Vietnam about handwashing with soap and water or the alcohol-based hand rub, and the associated factors during the COVID-19 pandemic in March 2020.

## 2. Material and methods

### 2.1. Study design

This was a cross-sectional study.

### 2.2. Study participant

Participants were eligible to participate if they were adults, currently lived in Vietnam, had internet connection, able to read, and understand the provided questions. Participants voluntarily participated in an online questionnaire administered through email and social networks.

### 2.3. Sample size and sampling

The study sample was calculated using the one-proportion sample size formula with an absolute precision *d* = 0.05, design effect *DE* = 2, and *p* was the proportion of handwashing people using soap at essential times (e.g., after going out). The calculated sample size was 277 people.

Participants were selected using non-probability, self-nominated sampling. The researchers sent the link contained online questionnaire to respondents through email, social networks (e.g., Facebook, Zalo, etc.). The online questionnaire was available from 0.00 am 25 March to 11.59 pm, 01 April 2020. At the time of the survey closing, 870 submissions were recorded. Among the submissions, 859 agreed to participate in the survey, making the response rate of 98.74%. Among those who agreed, 837 participants were included in the analysis, 22-excluded submissions were participants who had answered only 10% of total questions or fewer.

### 2.4. Data collection

We used Kobo Toolbox (kf.kobotoolbox.com) to develop the self-administered questionnaire and to collect data. The questionnaire could be easily accessed on any devices (e.g., computer, tablet, cellphones, etc.) with an internet connection through a link (https://ee.kobotoolbox.org/x/#Juy6CuzP). To prevent duplication, an email address was required for each time respondents submitting their responses. The average time to complete the questionnaire was approximately 10 minutes.

### 2.5. Measurements

The main dependent variable in this study was the correct reported practice of handwashing. It was a binary variable, which could be calculated by combining four following questions:

1. Did the respondent choose water and soap as a primary mean to handwashing whenever it was available? (2 available choices: Yes/No).

2. Could the respondent list ALL the 5 necessary times for handwashing? (2 available choices: Yes/No). Five mentioned necessary times were: 1) after going to the toilet, 2) after touching surfaces at public places (e.g.: elevator, doorknob, etc.), 3) before having meals, 4) before preparing meals for family and 5) after removing face mask.

3. Did the respondent have handwashing with 6 steps? (2 available choices: Yes/No).

4. Did the respondent have handwashing in at least 20 seconds? (2 available choices: Yes/No). (according to the United States Centers for Disease Control and Prevention recommendations [18]).

A participant could be considered having a correct reported practice of handwashing when he/she has answered Yes for all four above questions.

Independent variables were socio-economic information of respondents, including gender (male/female), age (in years), the highest educational level (high school or below, vocational/college/university), occupation (student/employee/other), marital status (single, married, separated/divorced/widowed)), a source where respondents get information about handwashing (friends, relatives, health staff, internet/social networks, newspaper/television, government/MOH), and knowledge of handwashing (in points). Knowledge variable was created through a combination of nine questions and the highest score would be 33, details were described as below:

**Table 1.** Details of questions regarding knowledge of handwashing.

No.	Question	Correct answer	Point
1	Diseases that could be spread by dirty hands	COVID-19, acute respiratory diseases, pneumonia, diarrhea, flu, helminth, hand-foot-mouth disease	Each selected disease: 1 point (max. 7 points) Other or do not know/do not remember: 0 point
2	Used soap/antiseptic solution when handwashing	Water and soap Antiseptic solution (with alcohol)	If each correct answer or both are selected: 1 point
3	Conditions which need handwashing	Before: having meals, cooking, feeding child After: leaving the toilet, childcare works, contacting patients or waste, touching pets	Each selected condition: 1 point (max. 8 points) Other or do not know/do not remember: 0 point
4	In COVID-19 pandemic, apart from mentioned above conditions, when handwashing should be done	After touching surfaces at public places After taking off face mask	Each selected choice: 1 point Other choices: 0 point
5	Necessary time for handwashing	At least 20 seconds At least 30 seconds	0.5 point 1 point
6	Describe taken steps when handwashing by water	6 steps of handwashing according to Vietnam MOH recommendations [19]	Each selected condition: 1 point (max. 6 points) Other or do not know/do not remember: 0 point
7	Frequency of each step when handwashing by water	5 times	1 point
8	Describe taken steps when handwashing by antiseptic solution (with alcohol)	6 steps of handwashing according to Vietnam MOH recommendations [19]	Each selected condition: 1 point (max. 6 points) Other or don't know/don't remember: 0 point
9	Frequency of each step when handwashing by antiseptic solution (with alcohol)	5 times	1 point

### 2.6. Date analysis and statistical method

Both descriptive and inferential statistics were performed in this study. All independent variables were described under the main outcome by calculating frequencies and percentages. Univariate linear regression was used for each independent variable with the main outcome with a significant level of 0.05. Multiple linear regression was conducted to provide a theoretical model with collected predictors.

### 2.7. Ethical considerations

This study was approved by the Institutional Review Board of the Hanoi University of Public Health under the Decision No. 105/2020/YTCC-HD3 dated 20 March 2020. The study information about objectives, selection criteria, personal information privacy, data protection, advantages, and potential harm, were all provided to participants before doing the online questionnaire. Each participant was assigned a study number, no private data was collected, and all collected data was coded.

## 3. Results

### 3.1. General information of respondents

The responses of 837 participants were included in the analysis. Out of 837 participants, 633 (75.7%) were female. The average age in years of the respondents was 33.3 (SD = 10.9), male respondents were older than females (35.7 vs. 32.6, respectively). Most respondents were employed, which accounted for about 60.5% while 25% of the respondents were students, and the rest of them were self-employed (4.4%), business/small business (3.8%), retired (2.8%), and other (2.3%). At the time of data collection, 23 of the63 provinces in Vietnam reported to have COVID-19 patients. In our survey, the proportion of respondents from the provinces with COVID-19 patients was about 72%. There was 64% of the respondents completing vocational/college/university as their highest educational level, while about 28% of total participants had completed postgraduate education. About half of the respondents were living with their spouse. The proportion of single respondents was 39.2%, and 3.8% of the respondents were separated/divorced/widowed.

**Table 2.** Characteristics of respondents.

Characteristics	Total (n = 837)		Males (n = 203)		Females (n = 634)	
Age in years, mean (SD)	33.3	10.9	35.7	11.8	32.6	10.4
Occupational status, n (%)						
Student	220	26.3	51	25.1	169	26.7
Employee	506	60.5	112	55.2	394	62.2
Self-employment	37	4.4	12	5.9	25	3.9
Business/small business	32	3.8	15	7.4	17	2.7
Retired	23	2.8	7	3.5	16	2.5
Other (e.g., farmer, housewife)	19	2.3	6	3.0	13	2.1
Provinces, n (%)						
Have COVID-19 patients	601	71.8	138	68.0	463	73.0
Do not have COVID-19 patients	236	28.2	65	32.0	171	27.0
Highest educational level, n (%)						
High school and below	63	7.5	23	11.3	40	6.3
Vocational/College/University	536	64.0	114	56.2	422	66.6
Postgraduate	234	28.0	65	32.0	169	26.7
Marital status, n (%)						
Single	328	39.2	69	34.0	259	40.9
Living with husband/wife	477	57.0	127	62.6	350	55.2
Separated/Divorced/Widowed	32	3.8	7	3.5	25	3.9

### 3.2. Handwashing behavior among respondents

Overall, the rate of respondents with correct reported practices of handwashing was 26.3%. When looking into four aspects that assessed respondents correct reported practices, using water and soap as a primary choice for hand hygiene was the aspect with the highest correct proportion, which accounted for almost 80% of the total respondents. In this survey, only 60.6% of the respondents had a correct answer for the all necessary times for handwashing, and 42.8% of the respondents could list all six steps of proper hand hygiene practices. The aspect in which the respondents had the lowest correct level was the adequate handwashing time. Only 28.4% of participants reported washing their hands in at least 20 seconds as recommended by WHO.

**Figure 1.**
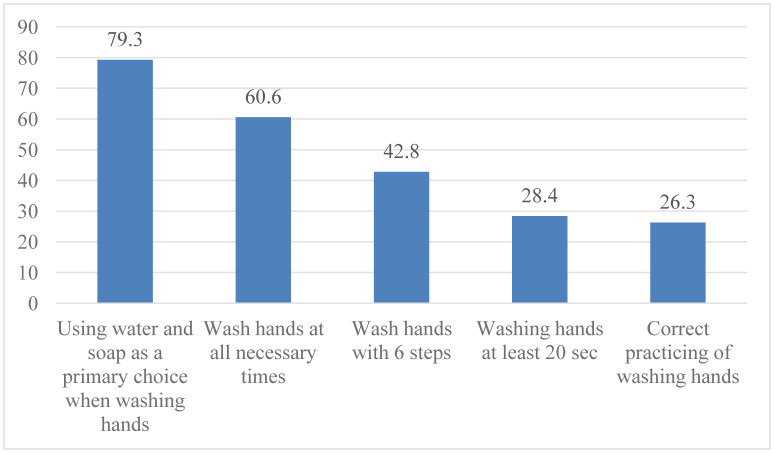
Respondents' reported practice of handwashing (n = 837).

Regarding the necessary time for handwashing, the popular times at which most respondents knew that they need to perform handwashing were: after going to the toilet (96.6%), after touching surfaces at places such as elevators, doorknobs, etc. (92.1%), and before having meals (92.1%). The proportion of respondents knew that they needed to wash hands before preparing meals for their families was 84.5% and only 69.3% of respondents answered that they needed to wash hands after removing their face masks.

**Figure 2.**
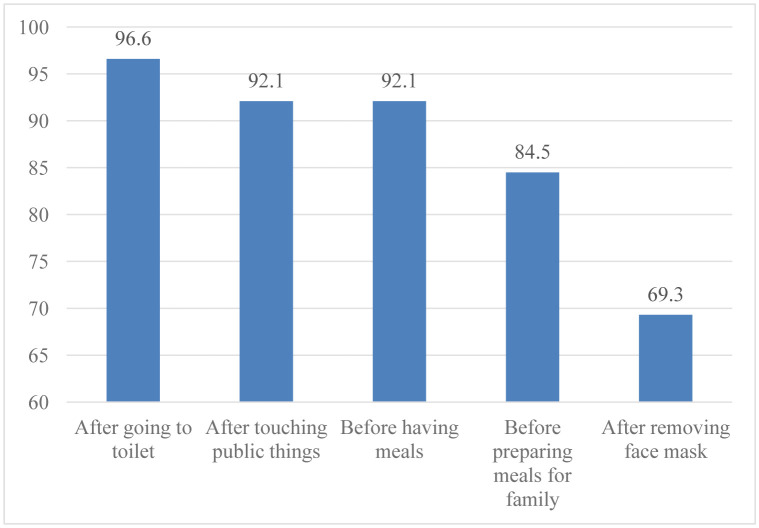
Respondents' information of necessary time for handwashing (n = 837).

### 3.3. Factors associated with reported handwashing practices of the participants

**Table 3.** Associations (both univariate and multivariate) between the reported practice of handwashing and respondent's characteristics.

Characteristics	Univariate analysis			Multivariate analysis		
	OR	95%CI	p-value	OR	95%CI	p-value
Age in years	1.02	1.01–1.04	0.002	1.02	0.99–1.05	0.24
Gender						
Male	Ref			Ref		
Female	1.55	1.06–2.28	0.024	1.88	1.15–3.09	0.012
Occupational status						
Student	Ref			Ref		
Employee	1.94	1.32–2.87	0.001	1.92	0.97–3.79	0.06
Other (e.g., small business, retired, farmer, housewife)	1.14	0.64–2.02	0.65	1.60	0.61–4.15	0.34
Provinces						
Do not have COVID-19 patients	Ref			Ref		
Have COVID-19 patients	0.81	0.58–1.14	0.22	1.21	0.77–1.90	0.40
Highest educational level						
High school or below	Ref			Ref		
Vocational/College/University	1.37	0.72–2.60	0.33	1.30	0.54–3.14	0.56
Postgraduate	1.51	0.77–2.96	0.23	1.26	0.48–3.28	0.64
Marital status						
Single	Ref			Ref		
Married	1.64	1.18–2.28	0.003	0.91	0.48–1.73	0.77
Separated/divorced/widowed	0.87	0.34–2.19	0.76	0.39	0.11–1.42	0.15
Getting information from:						
Friends	1.47	1.08–2.00	0.015	0.96	0.51–1.81	0.89
Relatives	1.31	0.96–1.79	0.09	1.09	0.58–2.06	0.78
Health staffs	3.03	2.05–4.47	<0.001	2.96	1.70–5.13	<0.001
Internet/social networks	0.78	0.48–1.25	0.30	0.44	0.21–0.96	0.04
Newspaper/Television	0.79	0.51–1.24	0.31	0.41	0.20–0.87	0.019
Government/MOH	2.16	1.20–3.87	0.01	1.64	0.76–3.53	0.21
Knowledge of handwashing (points)	1.34	1.25–1.43	<0.001	1.30	1.20–1.41	<0.001

Some characteristics were found to be statistically significant with reported practices of handwashing among the participants in the univariate analysis including age, gender, occupational status, marital status, getting COVID-19 information from friends, health staff, government/MOH, and knowledge of handwashing (in points). When adjusting all variables in multivariate analysis, female respondents were found likely to be 1.88 times (95% CI: 1.15–3.09) higher to have correct reported practices of handwashing than males. We also found that respondents who received information regarding handwashing from health staff had the odds of reported handwashing correctly 2.96 times (95% CI: 1.70–5.13) higher than those who did not. This survey also revealed the negative impacts of internet/social networks and newspaper/television on the reported practice of handwashing among the respondents. In detail, the participants who received hand hygiene information from the internet/social networks had 56% (95% CI: 4–79%) chances lower to wash hands correctly than those who did not. The same trend was observed among the respondents getting information from newspaper/television with a proportion of 59% (95% CI: 13–80%). At last, respondents who had more knowledge of handwashing correctly could have correct reported practices of this behavior, for each point increased in knowledge, the odds of reported practices correctly increased 1.3 times (95% CI: 1.2–1.41).

Our multivariate analysis did not found characteristics including age, occupational status, original provinces, educational level, marital status, getting information from friends, relatives, government/MOH to be associated with correct reported practices of handwashing among participants.

## 4. Discussion

### 4.1. Reported handwashing practices of the study respondents during the COVID-19 pandemic

The study results showed that the prevalence of people reported washing their hands with clean water and soap as their primary choice was remarkably high (79.3%). This result was relevant with the recommendation from the Vietnamese MOH that people should perform handwashing with soap and clean water. This method is considered as the priority measure for handwashing and the sterilized sanitizer is recommended only when soap and water are not available [20]. Our study found that participants' reported practices on handwashing (including handwashing with soap and with sterilized sanitizers) were rather high (60.6%) at essential times such as after defecation, before meals, before processing meals for family members, after contact with public places such as lifts, public corridors or doorknobs, and after removing masks during the time of the COVID-19 pandemic. This prevalence was significantly higher comparing to that during the non-pandemic time in which the prevalence of handwashing with soap and water among the community was reported to be very low at 10% after defecation [21]. The group of mothers of children under 5 years old is considered as a priority target group in all health communication programs in Vietnam, however, the handwashing practice rate among this group was only 24.3% at the essential times [22]. The remarkably high prevalence of reported handwashing of the community during the COVID-19 pandemic could be due in part to the fact that the Vietnamese Government did a very comprehensive communication strategy about COVID-19. The communication strategy included frequent delivery of information about the danger of COVID-19, its rapid transmission, and the prevention methods, including handwashing with soap and water, and with sterilized sanitizers to all mobile and Zalo users in Vietnam. As of January 2020, there were 145.8 million mobile users in Vietnam which is about 150% of the total population of the country. The number of internet users was 68.17 million, of whom, 74% used Zalo-the number three popular social media in Vietnam after Facebook and YouTube [23]. Therefore, it could be concluded that COVID-19 related information was disseminated to almost Vietnamese people. Also, mass media and the internet presented COVID-19 information at an exceedingly high frequency. These together might have contributed to the high prevalence of the reported handwashing practice. Another reason that may contribute to the explanation of the high frequency of reported handwashing was due to the unawareness of the community about the COVID-19, as it is the emerging infectious disease that happened in late 2019 in China. The unawareness might lead to the community's panic and outrage about the disease, especially when the number of cases surge daily, with 1,780,315 cases and 108,828 deaths in 211 countries and territories in the world at 12.00 pm of 12^th^ April 2020 [3]. According to Sandman (2012), the risk was characterized as hazard and outrage [24], therefore, people might have considered COVID-19 as a seriously dangerous disease and might have paid a lot of attention to the disease, including handwashing as a prevention measure.

However, the high frequencies of reported handwashing at essential times in this study might not have implied that the actual handwashing practices of the participants were that high, as many of the studies around the world have shown that the reported handwashing practices were much higher than observational handwashing behaviors [25],[26], even higher than 40 times in reported handwashing practices versus actual practices [25]. Therefore, the Vietnamese Government needs to regularly focus on the importance of handwashing with soap in prevention of COVID-19 disease and other infectious diseases transmitted via contaminated hands to maintain the high prevalence of handwashing at essential times, not only during the pandemic time but also in all the time, and therefore could contribute to the decrease of various preventable infectious diseases.

The study revealed that only 42.8% of the participants reported practicing all six steps during handwashing and only 28.4% performed hand hygiene for at least 20 seconds recommended by WHO [27], making the total prevalence of correct reported handwashing practice was only 26.3%. Although the Government had implemented comprehensive measures to prevent and control the disease, including the “Nationwide Social Distancing” Decree in 15 days, starting from 0.00 1^st^ April 2020 [28], this result implies that the intervention strategy of the Vietnamese Government to improve community's awareness and practices on COVID-19 prevention should also focus on how to guide the community to practice proper handwashing to effectively prevent of the spread of the disease in the community.

### 4.2. Factors associated with reported handwashing practices of the participants during the COVID-19 pandemic

It revealed from our study that female participants had better reported practices on handwashing than male participants (Table 3), which was consistent with other studies' results in Vietnam among child caretakers [22], a group of adolescents in Malaysia [29], and in an online survey in Hong Kong [30] although these studies did not collect data during the pandemic while our study was conducted during the COVID-19 pandemic.

Many international studies [31] and studies in Vietnam [22],[32] showed that educational level was an important factor affecting handwashing practices. However, a contrast result was obtained in our study. As discussed above, the frequent delivery of information about COVID-19 prevention and control measures to every mobile and Zalo users in Vietnam by the Vietnamese MOH and the Vietnamese Government could be a feasible reason to explain this result.

Besides, various studies showed that better knowledge associated with better reported handwashing practices [29],[33]. A similar trend was also observed in this study, with an increase of 1 knowledge score on handwashing contributed to the odds ratio of 1.3 (95% CI: 1.20–1.41) in reported handwashing practices. Intervention studies on sanitation and hygiene also confirmed that intervention to increase knowledge on handwashing would contribute to the increase in handwashing practices [21],[34].

One interesting finding from this study was that participants who obtained knowledge on handwashing from the internet sources (internet/social network) or newspapers/television had poorer handwashing practices that those who did not receive knowledge from these sources. The participants who gained knowledge from healthcare staff had better reported handwashing practices than those who did not get information from this staff. As suggested by Sandman (2012) above, due to the uncertain of the COVID-19 disease, the community's outrage for this disease increased and therefore contributed to the increased perception on the risk of the disease [24]. Based on the increased community outrage, fake news about the disease was quite common, especially on the internet and social media, which might have led to the poorer reported handwashing practices.

## 5. Limitations of the study

Although certain interesting results were found in this study, our study is subject to several limitations. First, our sample was not representative because we only collected data online, which might have resulted in most of the respondents were at higher educational levels (more than 90% had the college/university levels or higher). Other studies found that users of the internet were not representative of the general population, and not all felt familiar with the online protocol [35]. However, during the COVID-19 pandemic with social distancing suggestion and later ordered by the Government Decree on the 1^st^ of April 2020 [28], an online survey was the most convenient and feasible method for data collection. Secondly, all the study team members worked for a public health education institution, therefore, many of their social media friends might also interrelate to public health or medicine. This might lead to a higher chance of giving “right options” in the questionnaire as they know clearly interrelate to public health or medicine. We must accept this bias and consider it as a limitation for this study. Thirdly, due to the social distancing Decree of the Vietnamese Government, only online data collection was undertaken, and therefore we could only collect reported handwashing practices. This might have implied that the prevalence of reported handwashing practices might have much higher than the actual practice, as shown by various studies in the world [25],[26]. However, as explained earlier, due to the fear of acquiring COVID-19 during the pandemic, people tended to follow and undertake preventable measures, including handwashing practices [23], therefore these reported practices might have been relatively higher than that of the non-pandemic time. Last but not least, we did not collect data about respondents' income, place of living (urban, rural) in the data collection, the presence of handwashing places, and soap in their houses, although these variables are considered important indicators for handwashing practices. However, as we explained earlier, the data collection was undertaken during the COVID-19 pandemic, and handwashing with soap was not the only measure to be encouraged by the Vietnamese Government. Alcohol-based hand rub was also encouraged to be used to prevent COVID-19, especially when water and soap were not available. Therefore, we could only focus on collecting data by provinces/cities where there were COVID-19 patients and on educational messages that our respondents received from the MOH and the Government during the time of data collection.

## 6. Conclusion and recommendations

Although handwashing practices at all essential times recorded in this study was rather high, the prevalence of practiced washing hand correctly was low, mainly due to the incorrect practice on the six handwashing steps and the minimum required time for handwashing. The Government should pay attention to the communication strategy on COVID-19 prevention measures to the citizens, with a focus on the six steps of handwashing and the minimum required time for handwashing. Also, the Government should remind the Vietnamese people to select appropriate and reliable information on COVID-19 pandemic for references, especially information on the internet, social media, newspapers, etc.
